# MicroRNA-195 rescues ApoE4-induced cognitive deficits and lysosomal defects in Alzheimer’s disease pathogenesis

**DOI:** 10.1038/s41380-020-0824-3

**Published:** 2020-07-06

**Authors:** Jiqing Cao, Min Huang, Lei Guo, Li Zhu, Jianwei Hou, Larry Zhang, Adriana Pero, Sabrina Ng, Farida El Gaamouch, Gregory Elder, Mary Sano, Alison Goate, Julia TCW, Vahram Haroutunian, Bin Zhang, Dongming Cai

**Affiliations:** 1 James J Peters VA Medical Center, Research & Development, Bronx, NY 10468 USA; 2 Department of Neurology, Icahn School of Medicine at Mount Sinai, New York, NY 10029 USA; 3 Department of Genetics and Genomic Sciences, Icahn School of Medicine at Mount Sinai, New York, NY 10029 USA; 4 Mount Sinai Center for Transformative Disease Modeling, Icahn School of Medicine at Mount Sinai, New York, NY 10029 USA; 5 Cornell University, Ithaca, NY 14850 USA; 6 Department of Psychiatry, Icahn School of Medicine at Mount Sinai, New York, NY 10029 USA; 7 Alzheimer Disease Rsearch Center, Icahn School of Medicine at Mount Sinai, New York, NY 10029 USA; 8 Department of Neuroscience, Icahn School of Medicine at Mount Sinai, New York, NY 10029 USA; 9 Ronald M. Loeb Center for Alzheimer’s disease, Icahn School of Medicine at Mount Sinai, New York, NY 10029 USA; 10 James J Peters VA Medical Center, MIRECC, Bronx, NY 10468 USA

**Keywords:** Neuroscience, Diseases

## Abstract

Our recent findings link the apolipoprotein E4 (*ApoE4*)-specific changes in brain phosphoinositol biphosphate (PIP_2_) homeostasis to the susceptibility of developing Alzheimer’s Disease (AD). In the present study, we have identified miR-195 as a top micro-RNA candidate involved in the ApoE/PIP_2_ pathway using miRNA profiles in human ROSMAP datasets and mouse microarray studies. Further validation studies have demonstrated that levels of miR-195 are significantly lower in human brain tissue of *ApoE4*^+/−^ patients with clinical diagnosis of mild cognitive impairment (MCI) or early AD when compared to *ApoE4*^−/−^ subjects. In addition, brain miR-195 levels are reduced along with disease progression from normal aging to early AD, and cerebrospinal fluid (CSF) miR-195 levels of MCI subjects are positively correlated with cognitive performances as measured by mini-mental status examination (MMSE) and negatively correlated with CSF tau levels, suggesting the involvement of miR-195 in early development of AD with a potential impact on cognition. Similar differences in miR-195 levels are seen in *ApoE4*^*+/+*^ mouse hippocampal brain tissue and cultured neurons when compared to *ApoE3*^*+/+*^ counterparts. Over-expressing miR-195 reduces expression levels of its top predicted target *synaptojanin 1* (*synj1*), a brain PIP_2_-degrading enzyme. Furthermore, elevating miR-195 ameliorates cognitive deficits, amyloid plaque burden, and tau hyper-phosphorylation in *ApoE4*^*+/+*^ mice. In addition, elevating miR-195 rescues AD-related lysosomal defects in inducible pluripotent stem cells (iPSCs)-derived brain cells of *ApoE4*^*+/+*^ AD subjects while inhibiting miR-195 exacerbates these phenotypes. Together, our data uncover a novel regulatory mechanism of miR-195 targeted at *ApoE4*-associated brain PIP_2_ dyshomeostasis, cognitive deficits, and AD pathology.

## Introduction

The apolipoprotein E4 (*ApoE4*) allele has been identified as a major risk factor for Alzheimer’s Disease (AD) [1]. Numerous studies suggest that *ApoE4* effects Aβ clearance [2–7], neurofibrillary tangle burden [8–11], synaptogenesis, and synaptic plasticity [12–15], glial activation and neuro-inflammation [16–18]. Moreover, *ApoE* proteins play central roles in lipid metabolism and neuronal homeostasis [19]. Prior studies reveal distinct alterations in brain membrane phospholipid composition, metabolism, and selected enzyme activities in postmortem AD brains [20–24], that can be exacerbated by *ApoE4* [25]. We have reported that *ApoE* proteins are critical determinants of brain phosphoinositol biphosphate (PIP_2_) homeostasis, and the *ApoE4* isoform is dysfunctional in this process contributing to the increased susceptibility of cognitive decline in AD [26]. We have shown that brain PIP_2_ levels are lower in *ApoE4* brains and neurons due to the increased expression of a PIP_2_-dergading enzyme, synaptojanin 1 (synj1) [26].

The functional roles of PIP_2_ and synj1 have been implicated in AD pathogenesis by our laboratory and others [26–32]. For example, increased expression of synj1 is linked to early endosome enlargement [33], and *ApoE4*-associated cognitive deficits in AD [26]. The reduction of synj1 provides several beneficial effects in AD such as accelerating Aβ clearance via the lysosomal degradation pathway [30], ameliorating mild traumatic brain injury (TBI)-induced elevation in tau hyper-phosphorylation [31], and rescuing *ApoE4*-associated cognitive impairments [26]. However, molecular signaling mechanisms that link *ApoE4* with brain PIP_2_/synj1 pathways and impact on cognitive function remain elusive. We have previously shown that increased synj1 levels in *ApoE4* brains are partially due to a failure in efficient synj1 mRNA degradation [26]. mRNA stability is often regulated by micro-RNA (miRNA) binding to 3′-UTR regions of mRNA [34]. We postulate that brain synj1 expression may be differentially regulated by *ApoE* isoforms through miRNA modulation.

Several miRNAs have been previously implicated in various AD processes [35]. Moreover, changes in various brain miRNA levels between AD subjects and normal-aged controls have been reported [36, 37]. Therefore, in the present study, we leveraged the existing miRNA datasets from the Religious Orders Study and the Rush Memory and Aging Project (ROSMAP) [38, 39], in combination with miRNA array studies we performed in *ApoE4*^*+*^ and *ApoE4*^−^ cortical neurons. We have identified a miRNA, miR-195, as a top candidate that is differentially expressed between *ApoE4*^*+*^ and *ApoE4*^−^ carriers and targeted at *synj1* mRNA as predicted by multiple bioinformatics databases including mirDB [40, 41]. The changes in miR-195 levels are further validated using postmortem human and mouse brain tissue as well as cultured neurons. Furthermore, we have characterized a regulatory role of miR-195 in *ApoE4*-associated brain PIP_2_ dyshomeostasis, cognitive deficits, and AD pathology.

## Materials and methods

### Human miRNA expression profile and data preprocessing

The miRNA expression profile was downloaded from the ROSMAP study (Synapse 10.7303/syn3388564). miRNAs that had a call rate less than 95% and an absolute value of lower than 15 in <50% of the samples were removed. miRNA expression values were normalized using a variance stabilization normalization method. Cartridges were specified as batches and were corrected with the Combat function in the R package sva (V3.20.0). The data pre-processing resulted in 309 miRNAs in 511 samples. Pre-processed RNA-seq FPKM gene expression abundance data were also downloaded from the ROSMAP study (Synapse 10.7303/syn3388564). Genes with at least 1 FPKM in at least 10% of the samples were selected, and data were then corrected for confounding factors including batch, PMI, and RIN scores. The pre-processed gene expression profile contains 16,235 genes and 619 samples.

### Differential expression and miRNA-gene correlation analysis

Differential expression analysis was performed on all miRNAs between 111 *APOE4*^−/−^ (ε3/3) and 24 *APOE4*^*+/−*^ (ε3/4) carriers using the R package limma (V3.34.0) [42]. Multiple tests were adjusted using the Benjamini–Hochberg’s (BH) FDR method. Correlation analysis was performed between miRNAs and genes using spearman’s correlation test. The miRNA-gene correlation was also examined in each of the subgroups of AD diagnosis, sex, and *APOE* genotype.

### miRNA array studies of mouse primary neuron samples

Embryonic 17 days old *ApoE*^−/−^ cortical neurons were cultured for 7 days in vitro in the presence of conditioned media derived from *ApoE3* and *ApoE4* primary astrocyte cultures as described [26, 43] (*N* = 3/group). miRNAs were extracted using miRCURY extraction kits (Exiqon Inc.) and then labeled using miRCURY LNA microRNA Hi-Power Labeling kit, Hy3/Hy5, and hybridized on the miRCURY LNA microRNA Array. The quality assessment using control spike-in oligo nucleotides produced signals in expected range indicated successful labeling. Following normalization of quantified signals after background correction using global Lowess regression algorithm, unsupervised and supervised data analysis were performed. The microRNA profiling identified a subset of microRNAs that are differentially expressed in the *ApoE3* versus *ApoE4*-treated neurons.

### miRNA target prediction

Targets of the miRNAs were predicted with the R package multiMiR (V2.2.0), which is a miRNA–target interaction database complying nearly 50 million human and mouse data from 14 different databases [44].

### Human brain and CSF sample preparation

Equal amounts of postmortem human parietal cortex brain tissues (50 μg by net weight) from the NIH brain and tissue repository (NBTR), as well as cerebrospinal fluid (1 ml by net weight) from mild cognitive impairment (MCI) subjects enrolled in the James J Peters VAMC study (“Markers of Transition to AD in the Veterans with MCI”) and Icahn School of Medicine at Mount Sinai (ISMMS) AD Research Center participants were used for studies. The experimental procedures involving human sample handling were approved by the appropriate committees at James J Peters VA Medical Center (JJP VAMC) and ISMMS. Patient demographic information with N/group provided in Table S1.

### Animal models

Human *ApoE4*^*+/+*^ or *ApoE3*^*+/+*^ knock-in (KI) mouse models without [45–47] or with 5xFAD background [48, 49] were genotyped as described [50]. All animal experiments were performed in accordance with NIH guidelines and were approved by the JJPVAMC and ISMMS Institutional Animal Care and Use Committees (IACUC). Sex as a biological variable was taken into considerations with inclusion of both male and female mice in all experiments.

### Phospholipid analysis

Samples were subjected for lipid extraction, followed by the quantification by anion-exchange HPLC as described previously [30, 51].

### Mouse Neuronal and human iPSC culture

Primary cortical neurons were cultured as described [26, 43], before being fixed and stained for confocal microscopy analysis (Zeiss LSM) [52]. In some conditions, bovine serum albumin (BSA) or receptor-associated protein (RAP) was included in the cultured media (*N* = 3/group). Alternatively, cultured neurons were transfected with adeno-associated virus 2 (AAV2)-containing miR-195 or miR-374 or scramble controls for 5 days before subjected to analysis. The *ApoE4*^+/+^ and *ApoE3*^+/+^ iPSCs were differentiated into neural progenitor cells (NPCs) by dual SMAD inhibition followed by neural rosette selection and forebrain-specific patterning by 20 ng/ml FGF2 exposure as described [53, 54]. These NPCs were purified by MACS for CD271^−^/CD133^+^ [53] and differentiated to cortical neurons [55, 56] and a homogeneous population of astrocytes [54, 57] before subjected to viral transfection and confocal microscopy analysis. Alternatively, mouse cortical neurons derived from *ApoE4*^+/+^ and *ApoE3*^+/+^ KI mice with *synj1*^*+/+*^ or *synj1*^−/−^ genotypes were co-cultured with *ApoE4*^+/+^ and *ApoE3*^+/+^ iPSC-derived pure astrocytes. In some experiments, cultured neurons were transfected with AAV2-containing miR-195, scramble controls, or miR195 inhibitors that specifically prevent miR-195 binding to its target mRNA before co-culturing with iPSC-derived astrocytes. Cultured iPSC-derived neurons and astrocytes were then incubated with lysotracker red for various time periods before fixation and double-stained for pTau and a nuclear marker DAPI (blue) for confocal microscopy analysis (Zeiss). *N* = 4–5/condition.

### Stereotaxic injection and behavior studies

Eight to nine weeks old male and female *ApoE3*^*+/+*^ and *ApoE4*^*+/+*^ KI mice without 5xFAD background (*N* = 19–23/group), or with 5xFAD background (*N* = 15–17/group) were placed in the stereotaxic apparatus with AAV2 or scramble virus administered into the dorsal CA1 regions of bilateral hippocampal brain regions using pressure injection as described [58, 59]. Injection volumes (0.5–2.0 µl) were delivered over 10 min to avoid tissue damage. Six to nine months after viral delivery, mice were tested with the NOR task as described [26, 60, 61]. Mice were randomized for genotype and sex, and blinded throughout the behavior data collection and analysis, surgical manipulations, and sample collection followed NIH practice guidelines. Animals were excluded from behavior analysis if the total exploration time was less than 4 s or if they had an illness that prevented them from reliably completing the behavior tests.

### Brain and neuronal sample preparation and biochemical analysis

Snap-frozen mouse hemi-brains or cultured neurons were harvested in lysis buffer [62] and processed via step-wise solubilization [62, 63], followed by SDS-PAGE to determine levels of synj1, dyn1, holoAPP, and CTFs. Levels of Aβ_42_, Aβ_40_, pTau, Tau and ApoE were determined using high-sensitive ELISA kits. Some tissue was used for miRNA and RNA extraction followed by qPCR and RNA-seq analysis. Some animals underwent perfusion followed by brain tissue section for immunohistochemical staining of amyloid plaque, synj1 and pTau.

### Differential gene expression analysis for miR-195 treated mice

The RNA-seq samples collected from mouse brains were profiled on the Illumina HiSeq platform. Quality control of generated reads was performed using FASTQC (0.11.8). The raw sequencing reads were aligned to the GRCm38 mouse genome (release 95) using star aligner (V2.5.0b). Following the read alignment, gene expression was quantified at the gene level based on Ensembl gene model GRCm38.95 using FeatureCounts [64]. Genes with at least one count per million in all samples were considered as expressed and hence retained for further analysis. The trimmed mean of M-values normalization method [65] was used to adjust for sequencing library size differences. Differential expression analysis was then performed on the quality controlled and normalized gene expression data using the R package limma (V3.34.0). The comparisons were carried out between miR195-treated and scramble control samples stratified by sex and *APOE* genotype. Multiple tests were adjusted using the BH FDR method.

### Functional enrichment analysis

The functional enrichment analysis was carried out for genes significantly correlated with miRNAs in human ROSMAP dataset, predicted target genes of each miRNA of interest, and differentially expressed genes identified from miR195-treated mouse RNA-seq dataset. These genes were queried against the molecular signatures database (MSigDB v6.1) using Fisher’s Exact Test and gene set enrichment analysis (GSEA) [66].

### Antibodies and reagents

The anti-synj1 (rabbit polyclonal Ab, Novus, RRID: AB_11047653), anti-pTau AT8 and Tau-5 (ThermoFisher, RRID: AB_223647 and 10980631), anti-Rab5 (Santa Cruz Biotechnology, RRID:AB_628191), anti-β actin and tubulin (Santa Cruz Biotechnology, RRID:AB_476697 and 477498), anti-holoAPP MAB348 and 6E10 (Millipore RRID:AB_94882 and 564201), anti-beta-Amyloid (Cell Signaling Technology, RRID: AB_2056585), anti-MAP2 (Abcam, RRID:AB_297885), anti-dynamin clone 41 for (BD bioscience; RRID:AB_3976413), anti-mouse and rabbit HRP (ThermoFisher, RRID:AB_2556542 and 2540618), Texas-Red and Alexa_555_ conjugated anti-mouse and rabbit IgG (ThermoFisher, RRID:AB_10374713, 10983944, 2535987 and 1090271) were purchased. AAV2-containing miR-195, miR-374, scramble controls and miR-195 inhibitors were generated and obtained from ABM Inc. with detailed sequence information available (Am00100, Amm1017200 and Amm3026700). The miRNA extraction kit and qPCR probes for specific miRNAs were purchased from Exiqon Inc. The qPCR probes for *actin* (Hs1060665_g1), *synj1* (Hs00953234_m1 and Mm01210539_m1), *gapdh* (Mm99999915_g1), RNU6B (NR_002752), 18 s and 45 s (4331182, Mm03928990_g1) were also purchased from ThermoFisher.

### Statistical analysis

The sample sizes of each experiment were chosen based on power calculations derived from previous similar studies, which allowed us to determine group sizes needed to achieve statistically significant results. All experiments including controls were performed in randomly assigned groups. Sample collection and data analysis followed NIH practice guidelines. Experimenters were blinded to the experimental condition of the animal while conducting experiments. The conditions were revealed after quantification was completed. Levels of miR-195 and mi-374 were normalized to U6 and RNU6B (internal controls) while synj1 mRNA normalized to GAPDH and 18s, and then expressed as Log_2_fold of changes when compared to controls. Levels of synj1, dyn1, pTau, Tau, and holoAPP were normalized to β-actin levels and expressed as a percentage of the control. Absolute Aβ_42_, Aβ_40_, pTau, Tau, and ApoE concentrations were quantitatively determined by ELISA and expressed as a percentage of the control. Independent-samples *t-*tests were used to determine significant mean differences (the threshold for significance sets at *p* < 0.05). ANOVA with post-hoc tests were used to determine group differences for multiple comparisons. Pearson correlation coefficients were calculated to determine the linear relationship between the two variables. Equality of variance was checked for all statistical comparisons. When independent-samples *t*-tests were used and equality of variances of compared groups were not the same, the Welch’s corrections were applied. All statistical analysis was performed using Prism 8.0.

## Results

### MiR-195 is identified as a top candidate miRNA involved in *APOE*-regulated synj1 expression

**Fig. 1:**
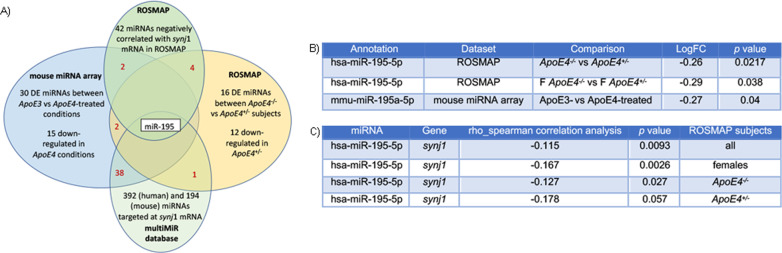
MiR-195 is identified as a top miRNA candidate involved in *APOE*-regulated synj1 expression. **a** Venn diagram shows miR-195 as the only miRNA in common shared among 4 groups: miRNAs differentially expressed between *ApoE4*^*+*^ and *ApoE4*^−^ carriers in the human ROSMAP dataset, miRNAs differentially expressed between *ApoE4*^*+*^ and *ApoE4*^−^ in the mouse miRNA array studies, miRNAs negatively correlated with *synj1* mRNA in ROSMAP, and miRNAs predicted to target at *synj1* mRNA by multiMiR database. Numbers of miRNAs overlapping among subgroups are indicated (red numbers). **b** Log Fold of changes (LogFC) and *p* values of differences in miR-195 levels between *ApoE4*^*+*^ and *ApoE4*^−^ carriers, between female *ApoE4*^*+*^ and *ApoE4*^−^ carriers in ROSMAP dataset, as well as differences in miR-195 levels between mouse *ApoE4*^*+*^ and *ApoE4*^−^ treated neurons. **c** Analysis of correlation between miR-195 and *synj1* mRNA in human subjects of the ROSMAP database.

First, we performed differential expression analysis between *ApoE4*^−/−^ (ε3/ε3; *N* = 111) and *ApoE4*^*+/*^^−^ (ε3/ε4; *N* = 24) carriers on human miRNA profiles in ROSMAP datasets. We identified 16 significantly differentially expressed miRNAs (*p* < 0.05), 12 of which with reduced expression in the *ApoE4*^*+/*−^ carriers (Fig. 1a). In parallel, we performed miRNA array studies of *ApoE*^−/−^ hippocampal neurons treated with *ApoE3* or *ApoE4*-conditioned media (CM) (Fig. 1a). We identified 30 significantly differentially expressed miRNAs (*p* < 0.05), 15 of which with reduced expression in the *ApoE4*-treated conditions (Fig. S1A). Among these miRNAs, miR-195 is the only differentially expressed miRNA between *ApoE4*^*+*^ and *ApoE4*^−^ conditions that is commonly shared between human and mouse datasets (hsa-miR-195-5p and mmu-miR-195a-5p; Fig. 1b). Another miRNA, miR-155 is also identified in both human and mouse datasets (hsa-miR-155 and mmu-miR-155-5p) but in opposite trends (higher in human *ApoE4*^*+/*−^ carriers and lower in mouse *ApoE4*-treated conditions; Fig. S1B).

Next, we performed prediction of miRNAs that putatively bind with *synj1* mRNA using 14 compiled multiMiR database [44]. From a total of 392 (human)/194 (mouse) miRNAs targeted at *synj1* mRNA (Fig. 1a), miR-195 was predicted as a top candidate in the human mirDB database [40, 41] (predicting score: 99.9/100). MiR-195 was also predicted as a top candidate miRNA targeted at *synj1* in several other databases (Fig. S1C) such as elmmo (predicting score: 0.80/1), and diana_micro (predicting score: 0.79/1).

Furthermore, we examined the correlation between *synj1* mRNA and all miRNAs in ROSMAP dataset using Spearman’s correlation test. MiR-195 is negatively correlated with *synj1* mRNA levels in all human subjects (Fig. 1c: *r* = −0.115, *p* = 0.0093). Similarly, negative correlations between miR-195 and *synj1* mRNA levels are seen in all female subjects (*r* = −0.167, *p* = 0.0026) and in *ApoE3*^*+/+*^ carriers (*r* = −0.127, *p* = 0.027). A negative correlation trend can be seen in *ApoE4*^*+/*−^ carriers as well but with no statistical significance (*r* = −0.178, *p* = 0.057), suggesting a possible weakened or perturbed network regulation between miR-195 and *synj1* in the presence of *ApoE4* allele. Separately, miR-374b also showed negative correlation with *synj1* mRNA (*r* = −0.09, *p* = 0.04). In addition, mmu-miR-374b-5p is differentially expressed between *ApoE3* and *ApoE4*-treated conditions but the differences in hsa-miR-374-5p levels between human *ApoE4*^*+/*−^ and *ApoE4*^−/−^ carriers are not statistically significant (Fig. S1B, *p* = 0.188).

To better understand the molecular pathways miR-195 regulates, we investigated the functions of predicted miR-195 targeted genes and those significantly correlated with miR-195 in the ROSMAP dataset. The top enriched functions for target genes and genes negatively correlated with miR-195 include regulation of neuronal and synaptic function, neurogenesis, and differentiation, while functions of genes positively correlated with miR-195 are enriched in the circulatory system and vasculature development (Table S2).

Together, these results support a role of miR-195 as a top candidate miRNA in regulating the ApoE-*synj1*-PIP_2_ pathways.

### Reduction of brain miR-195 levels is associated with *ApoE4* genotype, disease progression and cognitive decline

**Fig. 2:**
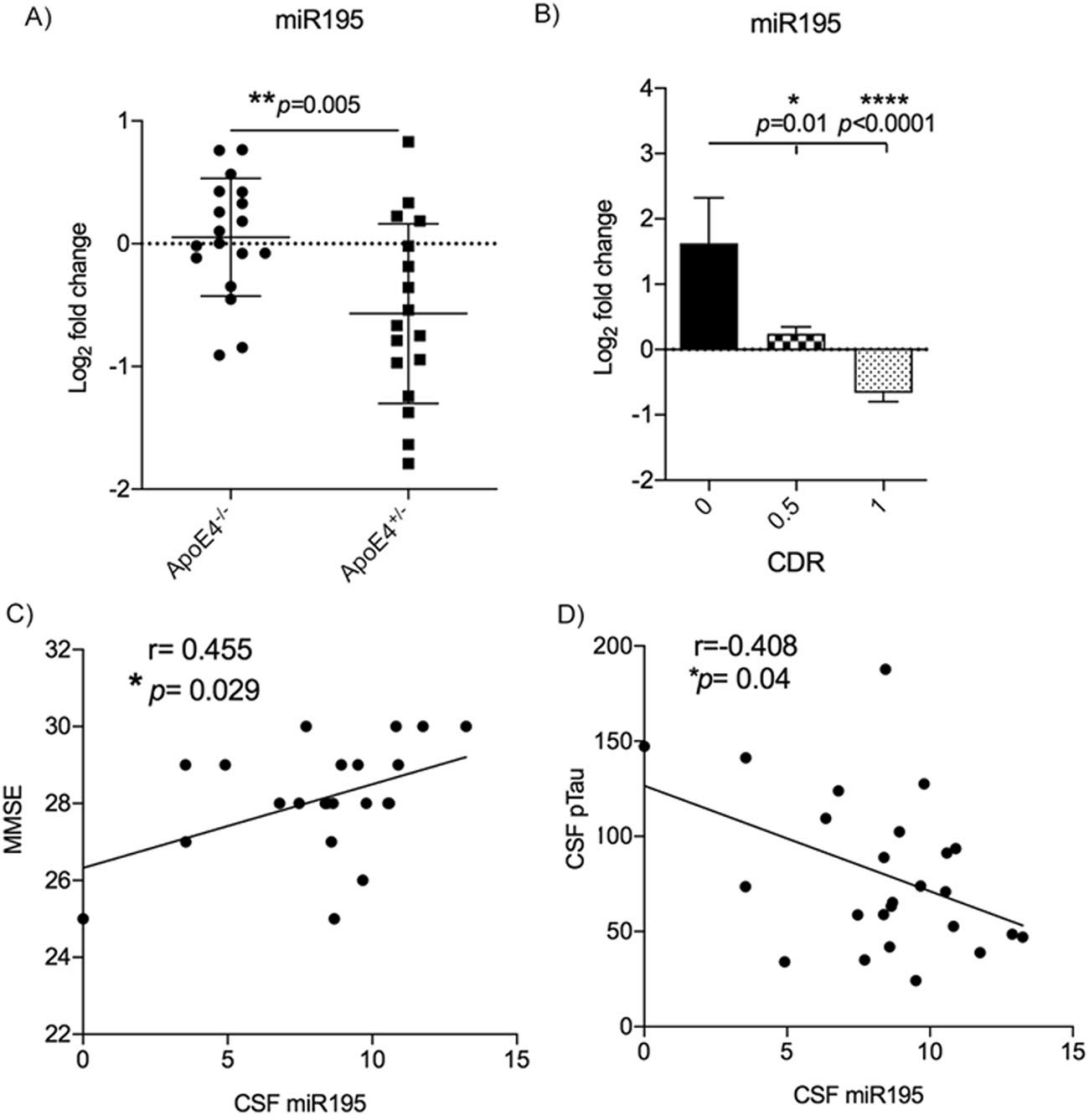
Reduction of brain miR-195 levels in human brain and CSF samples is associated with *ApoE4* genotype, disease progression, and cognitive decline. **a** Amounts of miR-195 (presented as Log_2_ fold changes) in human parietal cortex tissue of *ApoE4*^+/−^ subjects (CDR0.5-1) were lower than those in *ApoE4*^−/−^ subjects. *N* = 17–18/group; log_2_FC fold of changes: *ApoE4*^−/−^ 0.054 ± 0.113 versus *ApoE4*^+/−^ −0.570 ± 0.178, **p < 0.01 with independent-samples *t*-tests. **b** Pattern of reduction in miR-195 levels (presented as Log_2_ fold changes) along with AD disease progression from normal aging to MCI and early AD. *N* = 12–19/group; log_2_FC: 1.626 ± 0.696 in CDR 0 subjects versus 0.242 ± 0.104 in CDR 0.5 MCI patients; versus −0.663 ± 0.135 in CDR 1 AD subjects; *p < 0.05, ****p < 0.0001 with ANOVA tests. **c** Positive correlation between CSF miR-195 levels and MMSE scores (*r* = 0.455, *p* = 0.029; *N* = 23). **d** Negative correlation between CSF miR-195 and pTau levels (*r* = −0.408, *p* = 0.04; *N* = 23).

To validate differential expression patterns of miR-195 between *ApoE4*^*+/*−^ and *ApoE4*^−/−^ carriers, we next examined miR-195 levels in human brain tissue and CSF samples (subject demographic information provided in Table S1). We found that miR-195 levels were reduced in parietal cortex tissues derived from *ApoE4*^+/−^ mild cognitive impairment (MCI) and early AD subjects with clinical dementia rating (CDR) scores between 0.5 and 1 compared to levels in *ApoE4*^−/−^ donors (Fig. 2a). Interestingly, we observed a pattern of reduction in miR-195 levels along with disease progression from normal aging to MCI and early AD (Fig. 2b), similar to what we previously seen with PIP_2_ and phosphoinositol (PI) changes in early AD development [26]. We also found a significant reduction in miR-195 in female subjects compared to male subjects (Fig. S2A), with differences also noted between male *ApoE4*^−/−^ versus female *ApoE4*^+/−^ subjects. Consistently, we observed a reciprocal elevation of *synj1* mRNA levels in *ApoE4*^+/−^ subjects when compared to levels in *ApoE4*^−/−^ subjects (Fig. S2B). A positive correlation was noted between brain miR-195 and PIP_2_ levels in *ApoE4*^−/−^ carriers with CDR 0.5-1, and a positive correlation trend was seen in CDR 0-1 subjects regardless of *ApoE* genotypes (Fig. S2C). No correlation was seen between brain miR-195 and other phospholipid species, e.g., PI and phosphoinositol phosphate (PIP). No correlation was seen between brain miR-195 and other variables such as post-mortem interval (PMI) and age. A negative correlation was observed between brain miR195 and another known target of miR-195, beta-secretase 1 (BACE-1) [67] expression in the CDR 0.5–1 cohort (Fig. S2D). However, we did not observe any correlation between miR-195 and Aβ levels (data not shown).

There were statistically significant differences in human miR-374 levels between *ApoE4*^+/−^ and *ApoE4*^−/−^ subjects (hsa-miR374-5p; Fig. S3A). However, there were no significant changes in miR-374 levels along the disease progression except a transient elevation at MCI stage (Fig. S3A). Consistently with ROSMAP data (Fig. S1B), we also observed statistically significant differences in human miR-155 levels with higher levels in *ApoE4*^+/−^ subjects (hsa-miR155-5p; Fig. S3B). No significant differences were seen in miR-155 levels along disease progression (data not shown). Moreover, no significant differences were seen in miR-195 or miR-374 levels between *ApoE4*^+/−^ and *ApoE4*^−/−^ subjects of normal aging or advanced AD either (CDR 0 or 3 and above; data not shown), suggesting the functional involvement of miR-195 in early disease development and acceleration by *ApoE4* genotype.

Using cerebrospinal fluid (CSF) samples from a cohort of MCI subjects with CDR 0.5 (MCI defined by clinical examination and neuropsychological assessments), we found that CSF miR-195 levels were positively correlated with cognitive performance measured by mini-mental status examination (MMSE; Fig. 2c), and negatively correlated with pTau levels (Fig. 2d). While CSF PIP_2_ levels were below the detectable range, a positive correlation was seen between CSF cardiolipin and miR-195 (Fig. S3C), suggesting a potential involvement of miR-195 in mitochondrial function [68, 69].

Together, our results suggest that reduction of brain and CSF miR-195 levels is associated with *ApoE4* genotype, cognitive decline, and tau pathology during early AD development.

### MiR-195 expression is reduced in *ApoE4* mouse brains and cultured neurons

**Fig. 3:**
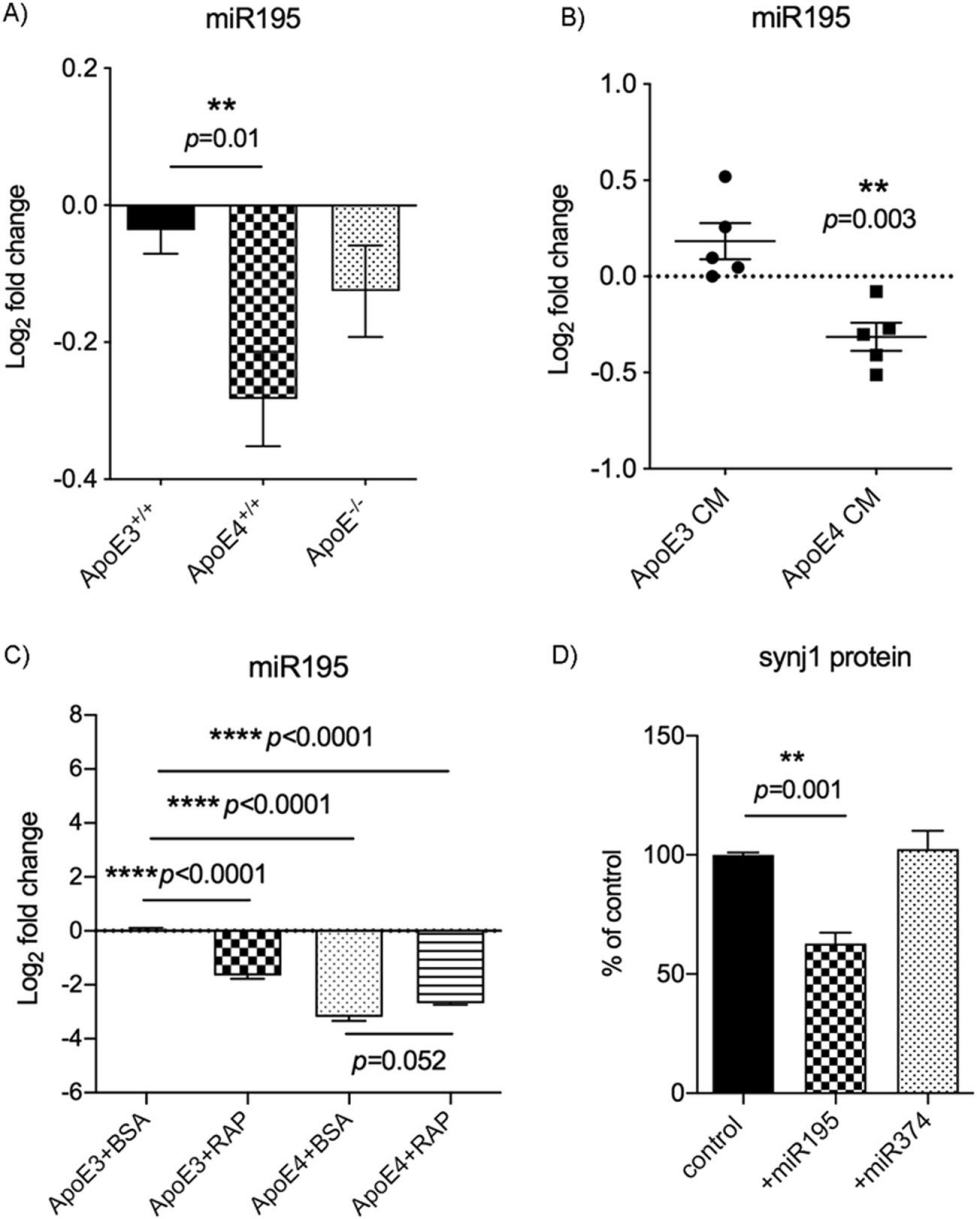
MiR-195 expression is reduced in hippocampal brain tissue and cultured primary neurons of *ApoE4* mice; modulating miR-195 levels regulates synaptojanin 1 expression. **a** Levels of miR-195 were reduced in 12-month old *ApoE4* hippocampal brain tissue (log_2_FC: −0.283 ± 0.069) when compared to those in *ApoE3* mice (log_2_FC: −0.036 ± 0.034). *N* = 11–13/group with both males and females; **p = 0.0096 with ANOVA tests. A nominal reduction in miR-195 levels was seen in *ApoE*^−/−^ brains with no statistical significance (log_2_FC: −0.125 ± 0.067, *p* = 0.48). **b** Levels of miR-195 in *ApoE*^−/−^ neurons treated with *ApoE4*-CM were reduced (log_2_FC = −0.314 ± 0.073,) when compared to levels of those treated with *ApoE3*-CM (log_2_FC: 0.184 ± 0.094). *N* = 5/group; **p = 0.003 with independent-samples *t*-tests. **c** Differences in miR-195 expression levels between *ApoE3*-CM and *ApoE4*-CM treated neurons were abolished in the presence of RAP. The treatment of RAP in the presence of *ApoE3*-CM led to a reduction in miR-195 levels (log_2_FC: −1.648 ± 0.125; *p* < 0.0001), whereas in *ApoE4*-CM treated conditions, miR-195 levels were much lower at baseline with a trend of improvement in the presence of RAP treatment (*ApoE4* CM + BSA log_2_FC: −3.193 ± 0.144 versus *ApoE4* CM + RAP log_2_FC: −2.678 ± 0.054; *p* = 0.052). *N* = 3/group; ****p < 0.0001 by One-Way ANOVA tests. **d** Synj1 protein levels were reduced with miR-195 over-expression but not miR-374 over-expression in *ApoE*^−/−^ hippocampal neurons in the presence of *ApoE4*-CM. *N* = 4/group; synj1 levels with miR-195: 62.87 ± 4.48% of controls, **p = 0.001; with miR-374: 102.4 ± 7.77% of controls, *p* = 0.93.

Next, we investigated if differences in miR-195 levels can be recapitulated in mouse models and primary neurons. We found that the levels of miR-195 were lower in 12-month old *ApoE4*^*+/+*^ mouse brains compared to *ApoE3*^*+/+*^ mice (Fig. 3a). A nominal reduction in miR-195 levels was seen in *ApoE*^−/−^ brains. Similarly, miR-374 was decreased in *ApoE4*^*+/+*^ mouse brains when compared to *ApoE3* conditions (Fig. S4A) with a nominal reduction in *ApoE*^−/−^ mice. In cultured *ApoE*^−/−^ hippocampal neurons, levels of miR-195 were consistently lower with *ApoE4* CM from astrocytes compared to those with *ApoE3* CM (Fig. 3b). A nominal difference was noted in miR-374 levels in neurons treated with *ApoE4* CM when compared to those treated with *ApoE3* CM (Fig. S4A) but failed to achieve statistical significance due to large variations among samples.

Interestingly, treatment of *ApoE*-receptor-associated protein (RAP), an inhibitor of *ApoE* receptors [70, 71] abolished differential expression patterns of miR-195 relative to control (BSA: bovine serum albumin). The RAP treatment in the presence of *ApoE3*-CM led to a reduction in miR-195 levels (Fig. 3c), whereas in *ApoE4*-CM treated conditions, miR-195 levels were much lower at baseline with a trend of improvement following RAP treatment. These results suggest that astrocyte-derived *ApoE* likely binds to *ApoE* receptors on neurons leading to changes in neuronal miR-195 and *ApoE4* exhibits loss-of-function effects on neuronal miR-195 expression.

We then determined if upregulation of miR-195 in *ApoE4* conditions could modulate expression levels of its predicted target gene, synj1. Over-expression of miR-195 but not miR-374 significantly reduced synj1 protein levels in *ApoE*^−/−^ neurons treated with *ApoE4* CM (Fig. 3d; synj1 levels with miR-195: 62.87 ± 4.48% of controls, *p* = 0.001; with miR-374: 102.4 ± 7.77% of controls, *p* = 0.93). No changes were seen in expression levels of another endocytic adapter protein dynamin 1 (dyn1; Fig. S4B), suggesting a specific effect of miR-195 on synj1 expression. Similarly, miR-195 over-expression in *ApoE3*^*+/+*^ or *ApoE4*^*+/+*^ neurons resulted in synj1 expression reduction in both mRNA and protein levels (Fig. S4C and D). It should be noted that *ApoE4*^*+/+*^ neurons exhibited more dramatic changes with over-expression of miR-195 in *synj1* mRNA (*ApoE3*^*+/+*^ w miR-195 log_2_FC: −1.084 ± 0.035 versus *ApoE4*^*+/+*^ w miR-195: −7.751 ± 0.043; Fig. S4C), and protein levels (*ApoE3*^*+/+*^ w miR-195 70.9 ± 21.2% versus *ApoE4*^*+/+*^ w miR-195 48.0 ± 9.84% of controls; Fig. S4D) than *ApoE3*^*+/+*^ neurons, possibly due to much lower baseline levels in *ApoE4*^*+/+*^ cells making them more sensitive to miR-195 manipulations.

### Over-expression of miR-195 rescues cognitive deficits and ameliorates AD-associated pathologies in *ApoE4* mouse models

**Fig. 4:**
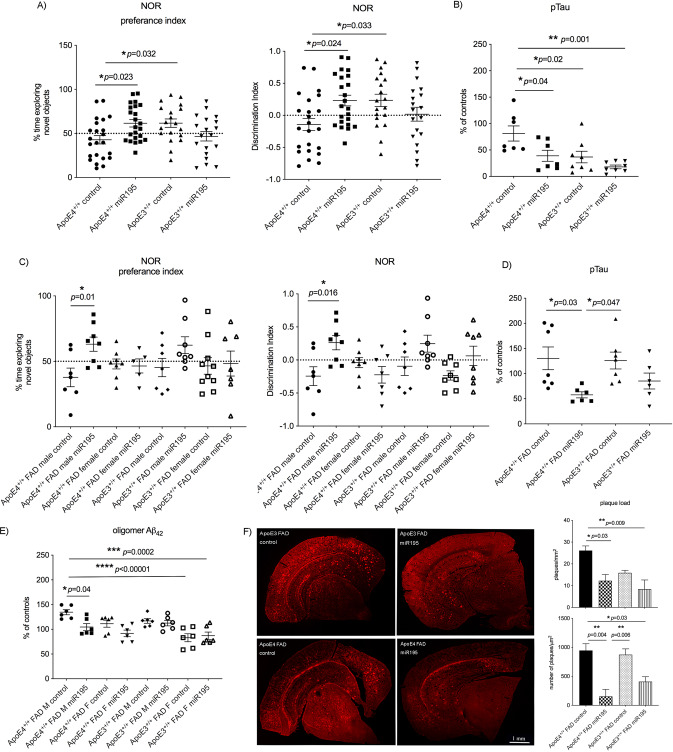
Over-expression of miR-195 rescues cognitive deficits and ameliorates AD-associated pathologies in *ApoE4* mouse models. **a** Preference index = (time exploring novel object)/(time exploring novel object + time exploring familiar object) and discrimination index = (time exploring novel object- time exploring familiar object)/(time exploring novel object + time exploring familiar object) in 4 groups of mice: *ApoE4*^+/+^ scramble injection, *ApoE4*^*+/+*^ miR-195 injection, *ApoE3*^*+/+*^ scramble injection, and *ApoE3*^*+/+*^ miR-195 injection. *N* = 19–23/group with both males and females; *p < 0.05 with ANOVA tests. **b** Levels of pTau in KI mouse hippocampus. *N* = 8/group with both males and females; *p < 0.05; **p < 0.01 with ANOVA tests. **c** Preference index and discrimination index in 8 groups of mice: *ApoE4*^+/+^ FAD male scramble injection, *ApoE4*^*+/+*^ FAD male miR-195 injection, *ApoE4*^+/+^ FAD female scramble injection, *ApoE4*^*+/+*^ FAD female miR-195 injection, *ApoE3*^*+/+*^ FAD male scramble injection, *ApoE3*^*+/+*^ FAD male miR-195 injection, *ApoE3*^*+/+*^ FAD female scramble injection, and *ApoE3*^*+/+*^ FAD female miR-195 injection. *N* = 6–10/group; *p < 0.05 with ANOVA tests. Levels of **d** pTau and **e** oligomer Aβ_42_ in EFAD mouse hippocampus. *N* = 6/group with both males and females; *p < 0.05; ***p < 0.001; ****p < 0.00001 with ANOVA tests. **f** Amyloid plaque burden in EFAD mouse hippocampus. A representative image of brain section is shown. Amyloid plaque load is quantified by density measured as area of plaques per mm^2^ of brain region, as well as total numbers of plaques/μm^2^ in 4 groups of mice: *ApoE4*^+/+^ FAD scramble injection, *ApoE4*^*+/+*^ FAD miR-195 injection, *ApoE3*^*+/+*^ FAD scramble injection, and *ApoE3*^*+/+*^ FAD miR-195 injection. *N* = 3/group; *p < 0.05; **p < 0.01 with ANOVA tests.

We next determined if over-expressing miR-195 could rescue *ApoE4*-associated cognitive dysfunction in vivo using *ApoE4*^*+/+*^ and *ApoE3*^*+/+*^ KI mice without and with AD transgenic background. We previously demonstrated that male human *ApoE4*^*+/+*^ KI mice manifested memory impairments as measured by novel object recognition (NOR) tests with an inability to discriminate between novel and familiar objects [26]. Here we found that *ApoE4*^*+/+*^ KI mice spent less time exploring novel objects than *ApoE3*^*+/+*^ KI mice did (Fig. 4a preference index: *ApoE4*^*+/+*^ versus *ApoE3*^*+/+*^ scramble controls: 42.9% versus 61.7%, *p* = 0.032), consistent with what we had previously observed [26]. This deficit was completely abolished by viral delivery of miR-195 bilateral hippocampi of *ApoE4*^*+/+*^ KI mice (Fig. 4a, 61.5%, *p* = 0.023 when compared to *ApoE4*^*+/+*^ scramble controls). However, no statistically significant differences were seen between scramble and miR-195 over-expressing *ApoE3*^*+/+*^ animals. Moreover, the discrimination index studies using the difference in exploration time for novel versus familiar object [72] showed consistent results suggesting that impaired discrimination behaviors in *ApoE4*^*+/+*^ KI mice were completely rescued by miR-195 over-expression (Fig. 4a discrimination index: *ApoE4*^*+/+*^ versus *ApoE3*^*+/+*^ scramble controls: −0.144 versus 0.234; *p* = 0.033; *ApoE4*^*+/+*^ scramble controls versus *ApoE4*^*+/+*^ miR-195: −0.144 versus 0.231; *p* = 0.024). The total amount of exploration time was comparable among all groups (data not shown).

Over-expressing miR-195 also reduced brain phospho-Tau (pTau) levels in *ApoE4* mouse brains (Fig. 4b; *ApoE4*^*+/+*^ scramble controls versus *ApoE4*^*+/+*^ miR-195: 81.3 versus 39.1% of controls; *p* = 0.04). Consistently, levels of *synj1* mRNA and protein levels were reduced in *ApoE4*^*+/+*^ mouse brains with miR-195 over-expression (Fig. S5A). Trends of reduction but to lesser degrees in pTau, *synj1* mRNA and protein levels were seen in *ApoE3*^*+/+*^ mice with miR-195 over-expression. No significant changes were seen in endogenous Aβ_40_, Aβ_42_ or ApoE levels with over-expression of miR-195 in *ApoE4*^*+/+*^ or *ApoE3*^*+/+*^ mouse brains (Fig. S5B and S5C). qPCR confirmed elevated miR-195 levels after viral manipulations (Fig. S5D).

Similar experiments were performed in *ApoE4*^*+/+*^ or *ApoE3*^*+/+*^ mouse models with 5xFAD background with AD-related pathological, neuro-inflammatory, and behavioral phenotypes manifesting at 4–8 months of age [48, 49]. Sex dimorphic responses were noted in these mouse models with male *ApoE4*^*+/+*^FAD mice being most sensitive to miR-195 manipulations (Fig. 4c preference index: *ApoE4*^*+/+*^FAD scramble controls versus miR-195: 37.8% versus 63.1%; *p* = 0.01; discrimination index: *ApoE4*^*+/+*^ scramble controls versus miR-195: −0.245 versus 0.262; *p* = 0.016). p-Tau reduction was also observed in *ApoE4*^*+/+*^FAD mice with miR-195 over-expression (Figs. 4d and S5E). Similar changes were seen in total Tau levels in *ApoE4*^*+/+*^FAD miR-195 over-expression mouse brains (data not shown). A dramatic reduction in brain oligomer Aβ_42_ measured by ELISA (Fig. 4e) and amyloid plaque burden determined by plaque numbers and plaque density (Fig. 4f) in *ApoE4*^*+/+*^ and *ApoE3*^*+/+*^FAD mouse brains was found with miR-195 over-expression. However, no significant changes were seen in levels of soluble Aβ_40_, Aβ_42_ (Fig. S5F), holo-APP or BACE-1 after miR-195 over-expression.

We also examined if miR-195 over-expression leads to changes in gene expression patterns and downstream pathways in EFAD mouse brains. Again, most differentially expressed genes (DEGs) are enriched in regulation of neuron, synapse, and immune functions (Table S3). Further GSEA studies [66] suggested top pathways perturbed by miR-195 over-expression are mitochondrial related pathways, consistent with studies in human brain dataset with top pathways enriched for genes negatively correlated with miR-195 involved in mitochondrial function (Table S2).

Together, our results suggest that elevating miR-195 levels in *ApoE4* mouse models without and with AD background can rescue *ApoE4-* and AD-related cognitive deficits and pathological changes.

### Over-expression of miR-195 endo-lysosomal defects in iPSC-derived brain cells of *ApoE4* AD subjects

**Fig. 5:**
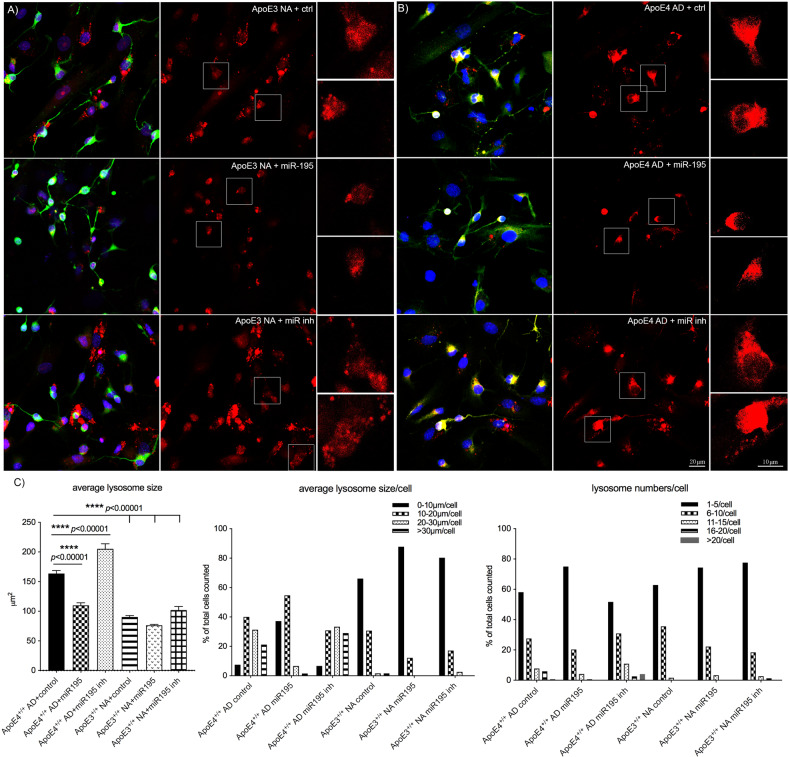
Over-expression of miR-195 rescues lysosomal defects in *ApoE4* iPSC-derived brain cells. Representative immunofluorescence co-staining of lysosomes (Lysotracker: red fluorescence), a neuronal marker MAP-2 (green fluorescence) and DAPI (blue fluorescence) of iPSC-derived neuron and astrocyte co-culture of (**a**) *ApoE3*^*+/+*^ normal aging (NA) and (**b**) *ApoE4*^*+/+*^ AD subjects with various conditions: scramble control (ctrl), miR-195, and miR-195 inhibitor (miR inh). Alternatively, *ApoE3*^*+/+*^ and *ApoE4*^*+/+*^ *N* = 3–6/conditions. **c** Quantification of all lysosomes by size (measured by areas; μm^2^) of 60–90 neurons (MAP-2^+^) in each experimental condition, the distribution of lysosome sizes/cell (measured by diameters; 0–10 μm, 10–20 μm, 20–30 μm and >30 μm), as well as the number of lysosomes in each cell (grouped by 1–5, 6–10, 11–15, 16–20, and >20 lysosomes/cell). ****p < 0.00001 with ANOVA tests.

We next investigated if manipulating miR-195 levels could ameliorate AD-related pathologies using human induced pluripotent stem cells (hiPSCs)-derived neuron and astrocyte co-culture from *ApoE4*^+/+^ AD subjects and *ApoE3*^+/+^ normal aging subjects (TCW et al., bioRxiv; 10.1101/713362). At baseline, *ApoE4*^+/+^ neurons (human iPSC or mouse) manifested enlarged lysosomes and increased numbers of lysosomes within each cell when compared to *ApoE3*^+/+^ counterparts (Fig. 5). The average size of lysosomes measured by area was 163.5 μm^2^ in *ApoE4*^+/+^ versus 90.4 μm^2^ in *ApoE3*^+/+^ neurons (Fig. 5c, *p* < 0.00001). There were 46.7% of *ApoE4*^+/+^ neurons with the diameter of lysosomes ranged from 10 to 20 μm, whereas 66.1% of *ApoE3*^+/+^ neurons with the diameter ranged between 0 and 10 μm. There were 18.4% *ApoE4*^+/+^ neurons with >10 lysosomes/cell, whereas only 1.6% *ApoE3*^+/+^ neurons with >10 lysosomes/cell. Over-expression of miR-195 in *ApoE4*^*+/+*^ neurons led to a significant reduction in lysosome size (109.9 μm^2^, *p* < 0.00001). The diameter of lysosomes and numbers of lysosomes per cell in *ApoE4*^+/+^ neurons after miR-195 over-expression (53.3% of neurons with the diameter ranged between 0 snd 10 μm; 1.7% with >10 lysosomes/cell) were also shifted toward the lysosomal phenotypes in *ApoE3*^+/+^ neurons at baseline. In contrast, treatment with a miR-195 inhibitor exacerbated the lysosomal phenotypes of *ApoE4*^*+/+*^ neurons (average size: 205.4 μm^2^, *p* < 0.00001) and increased numbers of lysosomes per cell (40% of neurons with the diameter of lysosomes >30 μm; 25% of cells with >10 lysosomes/cell). No significant differences were seen in *ApoE3*^+/+^ neurons treated with miR-195 over-expression or inhibition when compared to the baseline. The pTau levels were reduced with over-expressing miR-195 and increased with miR-195 inhibition as demonstrated by fluorescent staining (Fig. S6A) and ELISA (Fig. S6B).

MiR-195 levels were also lower in cultured iPSC-derived astrocytes from *ApoE4*^*+/+*^ AD subjects when compared to those in *ApoE3*^*+/+*^ normal aging (NA) iPSC-derived astrocytes (Fig. S6C, *p* = 0.002). The effects of miR-195 inhibitor on lysosomes can also be seen in *ApoE4*^*+/+*^ astrocytes with a significant increase in average size of lysosomes (Fig. S6D, control versus miR-195 inhibitor treatment 36.5 μm^2^ versus 67.7 μm^2^, *p* < 0.00001; Fig. S6E 8.3% of control astrocytes with the diameter of lysosomes >30 μm versus 17.6% of miR-195 inhibitor treated). However, no significant differences were seen in lysosomes of *ApoE4*^*+/+*^ astrocytes with miR-195 treatment when compared to control, or between *ApoE4*^*+/+*^ versus *ApoE3*^*+/+*^ astrocytes at baseline.

Similar experiments were performed using mouse *synj1*^*+/+*^ and *synj1*^−/−^ neurons co-cultured with *ApoE4*^*+/+*^ iPSC-derived astrocytes in the presence or absence of miR-195 over-expression. We found that genetic knockout of *synj1* manifested similar effects on lysosomal phenotypes as miR-195 over-expression. The average size of lysosomes was 101.5 μm^2^ in *synj1*^+/+^ versus 77.0 μm^2^ in *synj1*^−/−^ neurons (Fig. S6F, *p* < 0.00001). With miR-195 over-expression, the size of lysosomes was reduced to 75.8 μm^2^ in *synj1*^+/+^ neurons (*p* < 0.00001). However, over-expression of miR-195 in *synj1*^−/−^ neurons did not exhibit any additive effects (69.7 μm^2^), suggesting that miR-195 indeed acts through its target gene *synj1* to rescue AD-related lysosomal defects.

Together, these results suggest that elevating miR-195 levels in human iPSC-derived *ApoE4*^*+/+*^ AD brain cells can rescue lysosomal defects, whereas inhibiting miR-195 can exacerbate these phenotypes.

## Discussion

AD is a complex, multifactorial neurodegenerative process, and accumulating evidence indicates the importance of miRNAs in AD pathogenesis. Our studies characterize the functional involvement of a miRNA, miR-195 in *ApoE4*-associated pathology. Utilizing unbiased systems biology approaches as well as multiple computational prediction tools, we leverage existing human ROS-MAP miRNA datasets in combination with mouse micro-array studies and identify miR-195 as the top miRNA candidate in regulating the ApoE-*synj1*-PIP_2_ pathways and that this regulation is differentially modulated by *ApoE* genotypes. More importantly, our data reveal a novel regulatory role of miR-195 in the *ApoE4* genotype-associated cognitive and lysosomal defects that contribute to AD development.

Dysregulation in brain miRNAs has been described in human subjects and mouse models of AD with proposed involvement of Aβ-dependent and Aβ-independent pathways [36, 37, 73]. Our studies here demonstrate miR-195 reduction during AD development, which correlates with early disease progression but not with advanced stages of AD. *ApoE4* genotype accelerates miR-195 reduction, which coincides with cognitive decline and increased tau pathology (Fig. 2). Previously, we reported patterns of changes in phosphoinositol (PI) metabolites correlated with disease conversion from normal aging to early AD [26]. Our results further indicate miR-195 changes are consistent with alternations in brain PIP_2_ levels (Fig. S2C). These findings highlight the potential of utilizing miR-195 levels as surrogate biomarkers to monitor brain PIP_2_ homeostasis and cognitive performance and detect early AD development and progression. Our studies of differentially expressed miRNAs between *ApoE4*^*+/*−^ and *ApoE4*^−/−^ carriers in ROSMAP and mouse microarray datasets also identified another miRNA in common, miR-155. A prior report suggested a protective role of miR-155 in AD with increased brain miR-155 levels in aged AD transgenic mice (APP/PS1 or Tau Tg) and in AD human subjects compared to levels in controls [73]. On the other hand, our data show that miR-155 is up-regulated in human *ApoE4*^*+/*−^ carriers but down-regulated in mouse ApoE4 condition (Fig. 1c). Nevertheless, the involvement of miR-155 in *ApoE4* pathogenic function remains unclear with opposing changes in human and mouse datasets.

Interestingly, functional enrichment studies of human dataset (Table S2) and mouse transcriptomic dataset (Table S3) implicate the roles of miR-195 in regulating neuronal and synaptic function, neurogenesis, and differentiation. Consistently, our data indicate that restoring miR-195 levels in vivo rescues *ApoE4*-associated cognitive deficits (Fig. 4a, c), ameliorates amyloid plaque burden (Fig. 4e, f) and pTau levels (Fig. 4b, d), and improves lysosomal defects in cultured human iPSC-derived brain cells (Fig. 5). While we did not see any reduction in endogenous mouse Aβ in ApoE KI mouse models (Fig. S5B) or soluble Aβ levels in EFAD mice (Fig. S5F), we observed dramatic decreases in oligomer Aβ levels and plaque burden with over-expression of miR-195 (Fig. 4e, f). In addition, we found a negative correlation between miR-195 and BACE1 expression in human brain tissue (*r* = −0.516, *p* = 0.004; Fig. S2D), consistent with a previous report [67]. However, no changes in BACE-1 levels were seen with miR-195 over-expression (data not shown). Together, these data suggest that miR-195 most likely regulates Aβ clearance instead of Aβ generation, and restoration of lysosomal function may facilitate these processes, as indicated in our prior reports that synj1 reduction accelerates lysosomal clearance of Aβ [43].

Moreover, a functional role of miR-195 in regulating pTau levels is implicated (Fig. 4 and Fig. S6A and S6B), consistent with our recent findings that down-regulation of synj1 prevents mild TBI-induced tau hyper-phosphorylation [31]. It would be interesting to further investigate the molecular mechanisms by which miR-195 regulates tau hyper-phosphorylation. It was previously reported that the exosomal secretion of tau may play an important role in tau spread, which could be regulated by miRNAs [74]. We speculate that miR-195 might serve as the key in modulating tau pathology secondary to impaired clearance through the lysosomal pathway and/or accelerated spread through the exosomal secretory pathway.

The results suggest that differential regulation of miR-195 expression by *ApoE* isoforms is mediated through binding to *ApoE* receptors on neurons, and the *ApoE4* genotype loses the ability to regulate miR-195 levels (Fig. 3c). In addition, our data showing *ApoE*^−/−^ with lower miR-195 expression like *ApoE4* (Fig. 3a), further supports loss-of-function effects of *ApoE4* on miR-195 expression leading to increased *synj1* mRNA and protein expression. Over-expression of miR-195 in *synj1*^−/−^ neurons failing to exhibit any additive effects on lysosomal enlargement (Fig. S6F) further strengthens the concepts that miR-195 rescues AD-related phenotypes through its target gene, *synj1*. It should be noted that *ApoE4*^*+/*−^ carriers exhibit higher sensitivity to miR-195 manipulations than *ApoE4*^−/−^ subjects (Figs. 4 and S5; Figs. 5 and S6), possibly due to much lower baseline miR-195 levels. Furthermore, differences in miR-195 levels between *ApoE4*^*+/*−^ and *ApoE4*^−/−^ conditions are not only seen in neurons but also in other brain cells such as astrocytes (Fig. S6C). The cell-type specific changes in miR-195 may contribute to different aspects of disease pathogenesis. We speculate that reduction in neuronal miR-195 levels may contribute to cognitive and synaptic dysfunction, while reduction in astrocytic miR-195 levels may play a role in defects in the secretory pathways leading to accelerated tau accumulation and spread.

We also examined sex impact in miR-195 expression. In ROSMAP dataset, the differences in miR-195 levels between *ApoE4*^*+/*−^ and *ApoE4*^−/−^ carriers persist in female subjects (Fig. 1b), similarly to our previous report of sex-specific effects on brain PIP_2_ homeostasis [26]. Sex dimorphism is also noted in miR-195 expression with much lower levels in female subjects, particularly in *ApoE4*^−/−^ carriers (Fig. S2A). EFAD mice also exhibited sex dimorphic responses to miR-195 manipulations with improved cognitive function and reduced oligomer Aβ levels in male but not female EFAD mice (Fig. 4c, e). Age-related changes in miR-195 expression and *synj1* mRNA levels have also been noted in *ApoE* KI and EFAD mouse brains, with differential expression between *ApoE4*^*+/+*^ and *ApoE4*^−/−^ mice more prominent at 12 months of age compared to a younger age, whereas differences in miR-195 in EFAD mice are already evident at 4 months of age (data not shown), suggesting that *ApoE*-genotype associated miR-195 changes might be exacerbated by aging and/or manifestations of AD pathologies.

While AD manifests as a multi-faceted disease process, targeting a specific miRNA to restore dysregulated networks and pathways at multiple levels could provide a promising avenue for future drug development. Therapeutic strategies directed at *ApoE4* have been and are actively explored in several preclinical and clinical studies such as immunotherapies, antisense oligonucleotide treatments, gene editing, modulators of *ApoE* expression, as well as small molecules to enhance *ApoE* lipidation, to correct its structures, to compete receptor binding, and to inhibit *ApoE*-Aβ interaction [75, 76]. Our findings here suggest a novel therapeutic direction that modulates *ApoE4* pathogenic function by a miRNA miR-195 through brain PIP_2_ lipid signaling pathways with multiple beneficial effects besides impact on Aβ and tau pathology.

In summary, our studies provide a mechanistic link between *ApoE4* genotype-specific changes in brain miR-195 expression with AD-related phenotypes including brain phospholipid dysregulation, cognitive deficits, lysosomal defects, and tau pathologies. These studies may uncover novel therapeutic strategies targeted at a specific miRNA miR-195.

## Supplementary information

Supplemental Figure Legends

Supplemental Figure 1

Supplemental Figure 2

Supplemental Figure 3

Supplemental Figure 4

Supplemental Figure 5

Supplemental Figure 6

Supplemental Table 1

Supplemental Table 2

Supplemental Table 3

## Data Availability

The human miRNA and RNA-seq datasets from ROSMAP studies are available via the AD Knowledge Portal (https://www.synapse.org/#!Synapse:syn5856115 and https://www.synapse.org/#!Synapse:syn3505720). The AD Knowledge Portal is a platform for accessing data, analyses, and tools generated by the Accelerating Medicines Partnership (AMP-AD) Target Discovery Program and other National Institute on Aging (NIA)-supported programs to enable open-science practices and accelerate translational learning. Data are available for general research use according to the following requirements for data access and data attribution (https://adknowledgeportal.synapse.org/DataAccess/Instructions). The mouse microarray studies and RNA-seq datasets after miR-195 perturbation experiments generated in this paper have been deposited to the AD Knowledge Portal as well (https://www.synapse.org/#!Synapse:syn22123695).
