# Supplementary material for: MicroRNA-195 rescues ApoE4-induced cognitive deficits and lysosomal defects in Alzheimer’s disease pathogenesis
Source: Mol Psychiatry. 2020 Jul 6;26(9):4687–701. doi: 10.1038/s41380-020-0824-3 (PMC7785685; doi:10.1038/s41380-020-0824-3)
Supplement: Supplementary file 1 — Supplemental Figure Legends [file 41380_2020_824_MOESM1_ESM.docx]

**Supplemental Figure Legends**

**Supplemental Figure 1. MiR195 is identified as a top miRNA candidate involved in *APOE*-regulated synj1 expression.** A) Among 30 differentially expressed miRNAs between *ApoE3* or *ApoE4*-conditioned media (CM) treated *ApoE*^-/-^ hippocampal neurons in microarray analysis, 15 are down-regulated in *ApoE4* conditions. B) Another miRNA, miR-155, is differentially expressed between *ApoE4^+^* and *ApoE4^-^* carriers that is commonly shared between human ROSMAP dataset and mouse miRNA array studies, but miR-155 levels change opposite trends (higher in human *ApoE4^+/-^* carriers and lower in mouse *ApoE4*-treated conditions). miR-374 is differentially expressed between *ApoE4^+^* and *ApoE4^-^* carriers in mouse miRNA array studies (*p*=0.0485) but not in the human ROSMAP dataset (*p*=0.188). C) Predicting scores of miR-195 targeted at *synj1* mRNA using human and mouse multiMiR database.

**Supplemental Figure 2. Reduction of brain miR-195 levels in human brain and CSF samples is associated with *ApoE4* genotype, disease progression, and cognitive decline.** A) Significant reduction in miR-195 levels in female subjects were seen compared to male subjects (log_2_FC: 0.284±0.141 in male subjects versus -0.343±0.123 in female subjects, *p*=0.008), with differences also noted between male *ApoE4*^-/-^ subjects versus female *ApoE4*^+/-^ subjects (log_2_FC: 0.340±0.120 in male *ApoE4*^-/-^ subjects *versus* -0.598±0.175 in female *ApoE4*^+/-^ subjects, *p*=0.02). B) Reciprocal elevation of *synj1* mRNA levels was seen in *ApoE4*^+/-^ subjects when compared to levels in *ApoE4*^-/-^ subjects (log_2_FC: *ApoE4*^-/-^ 1.073±0.286 *versus* *ApoE4*^+/-^ 2.093±0.310, *p*=0.02). C) Positive correlation between brain miR-195 and PIP_2_ levels was seen in *ApoE4*^-/-^ carriers with CDR 0.5-1 (*r*=0.472 *p*=0.048; N=18) with a positive correlation trend in CDR 0-1 subjects regardless of *ApoE* genotypes (*r*=0.283 *p*=0.06). D) Negative correlation between brain miR195 and BACE-1 expression was seen in the CDR 0.5-1 cohort (*r*=-0.52 *p*=0.004).

**Supplemental Figure 3. Reduction of brain miR-195 levels is associated with *ApoE4* genotype, disease progression and cognitive decline in human brain and CSF samples.** A) Amounts of miR-374 in human parietal cortex tissue of *ApoE4*^+/-^ subjects (CDR0.5-1) were lower than those in *ApoE4*^-/-^ subjects. N=17-18/group; log_2_FC: *ApoE4*^-/-^ 0.285±0.105 *versus* *ApoE4*^+/-^ -0.306±0.171, ***p*<0.01 with independent-samples t-tests. However, along the disease progression, there was a transient elevation in miR-374 levels at MCI stage but no significant differences were seen between CDR 0 (normal aging) and CDR1 (early AD) cohorts (log_2_FC: -1.160±0.830 in CDR0 subjects *versus* 0.299±0.098 in CDR 0.5 MCI patients, *p*=0.04). B) Higher miR-155 levels seen in *ApoE4*^+/-^ subjects (log_2_FC: *ApoE4*^-/-^ -2.275±0.280 *versus* *ApoE4*^+/-^ -1.107±0.451, *p*=0.035). C) Positive correlation was seen between CSF miR-195 and cardiolipin levels (r=0.684, *p*=0.0003; N=23).

**Supplemental Figure 4. MiR-195 expression is reduced in hippocampal brain tissue and cultured primary neurons of *ApoE4* mice; modulating miR-195 levels regulates synaptojanin 1 expression.** A) Levels of miR-374 were reduced in 12-month old *ApoE4* hippocampal brain tissue (Log_2_FC -0.455±0.098) when compared to those in *ApoE3* mice (Log_2_FC -0.026±0.050, *p*=0.02). A nominal reduction in miR-374 levels were seen in *ApoE*^-/-^ mouse brains but without statistical significance (Log_2_FC -0.197±0.124, *p*=0.40). N=8-10/group. A nominal difference was noted in miR-374 levels in neurons treated with *ApoE4* CM (log_2_FC: -0.037±0.245) when compared to those treated with *ApoE3* CM (log_2_FC: 0.328±0.254; *p*=0.33) but without statistical significance due to large variations among samples. N=5/group. B) No changes in dyn protein levels were seen in neurons over-expressing miR-195 or miR-374 (miR-195 over-expression 97.7% of controls; miR-374 over-expression 107.2% of controls). N=4/group. A representative example of western blot studies is shown. C) Over-expression of miR-195 in *ApoE3^+/+^* or *ApoE4^+/+^* neurons reduced synj1 mRNA levels. *ApoE4^+/+^* neurons exhibited more dramatic changes in *synj1* mRNA levels with over-expression of miR-195 when compared to *ApoE3^+/+^* (*ApoE3^+/+^* w miR-195 log_2_FC: -1.084±0.035 versus *ApoE4^+/+^* w miR-195 log_2_FC: -7.751±0.043). *****p*<0.0001 with independent-samples t-tests. D) Over-expression of miR-195 in *ApoE3^+/+^* or *ApoE4^+/+^* neurons reduced synj1 protein levels. Again, *ApoE4^+/+^* neurons exhibited more dramatic changes in synj1 protein levels with over-expression of miR-195 when compared to changes in *ApoE3^+/+^* neurons (*ApoE3^+/+^* w miR-195 70.9±21.2% versus *ApoE4^+/+^* w miR-195 48.0±9.84% of controls). ***p*=0.01 with independent-samples t-tests.

**Supplemental Figure 5. Over-expression of miR-195 rescues cognitive deficits and ameliorates AD-associated pathologies in *ApoE4* mouse models.** A) Levels of *synj1* mRNA and protein levels are reduced in *ApoE4^+/+^* mouse brains with miR-195 over-expression. *ApoE4^+/+^* scramble controls *versus* *ApoE4^+/+^* miR-195: *synj1* mRNA Log_2_FC 0.52 *versus* -1.29; ***p*=0.0005. synj1 protein 77.7 versus 56.4% of control; ***p*=0.006. Trends of reduction with lesser degrees in *synj1* mRNA and protein levels were seen in *ApoE3^+/+^* mouse brains with miR-195 over-expression. B) No significant changes in endogenous mouse Aβ_40_ or Aβ_42_ levels with over-expression of miR-195 in *ApoE4^+/+^* or *ApoE3^+/+^* brains were observed. C) No significant changes in ApoE levels with over-expression of miR-195 in *ApoE4^+/+^* or *ApoE3^+/+^* mouse brains were present. However, ApoE levels are much higher in *ApoE3^+/+^* mouse brains after miR-195 over-expression when compared to those in *ApoE4^+/+^* control or miR-195 injection mice. **p*<0.05 with ANOVA tests. D) Elevated miR-195 levels in both *ApoE4^+/+^* and *ApoE3^+/+^* mouse brains after viral manipulations are confirmed by qPCR. **p*<0.05, *****p*<0.00001 with ANOVA tests. E) Representative example of western blot analysis of pTau, total Tau, synj1 protein, and β-actin in E4FAD and E3FAD mouse brains without or with miR-195 manipulation is shown. F) No significant changes are seen in soluble Aβ_40_ and Aβ_42_ levels in E4FAD and E3FAD mouse brains without or with miR-195 manipulation. However, E4FAD mouse brains exhibit higher levels of soluble Aβ_40_ when compared to levels in E3FAD mice regardless of miR-195 manipulation.

**Supplemental Figure 6. Over-expression of miR-195 rescues lysosomal defects in *ApoE4* iPSC-derived brain cells.** A) Representative example of pTau staining (AT8) in iPSC-derived neuron and astrocyte co-culture after control, miR-195 or miR-195 inhibitor transfection. Quantification of immunofluorescence intensity shown in bottom panels. **p*<0.05 with ANOVA tests. B) Western blot analysis of pTau and synj1 protein levels of iPSC-derived brain cell culture from *ApoE3^+/+^* normal aging (NA) and *ApoE4^+/+^* AD subjects with scramble control (ctrl) or miR-195 transfection. C) MiR-195 levels in cultured iPSC-derived astrocytes from *ApoE3^+/+^* normal aging (NA) and *ApoE4^+/+^* AD subjects. ***p*<0.01 with independent-samples t-tests. D) Representative examples of immunofluorescence co-staining of a neuronal marker MAP-2 (red fluorescence), an astrocyte marker GFAP (green fluorescence) and DAPI (blue fluorescence), as well as immunofluorescence co-staining of an astrocyte marker GFAP (green fluorescence), lysosomes (Lysotracker: red fluorescence) and DAPI (blue fluorescence) of iPSC-derived neuron and astrocyte co-culture. Quantification of all lysosomes by size (measured by areas; μm^2^) of 60-90 astrocytes (GFAP^+^) in each experimental condition. **p*<0.05, ****p*<0.001, *****p*<0.00001 with ANOVA tests. E) Quantification of the distribution of lysosome sizes per astrocyte (measured by diameters; 0-10μm, 10-20μm, 20-30μm and >30μm), as well as the number of lysosomes in each astrocyte (grouped by 1-5, 6-10, 11-15, 16-20 and >20 lysosomes/cell). F) Quantification of all lysosomes by size (measured by areas; μm^2^) of 70-100 neurons (MAP2^+^) in each experimental condition: synj1^+/+^+control, synj1^+/+^+miR-195; synj1^-/-^+control, and synj1^-/-^+miR-195. *****p*<0.00001 with ANOVA tests.

**Supplemental Table 1. Sample information for human brain tissue cohorts.** The sample size and characteristics of subjects in the CDR 0-3 with *ApoE4*^-/-^ and *ApoE4*^+/-^ genotypes are shown (PMI, post-mortem interval). The PMI was comparable between groups.

**Supplemental Table 2. Functional analysis of genes negatively or positively correlated with miR-195 as well as predicted target genes of miR-195.** The top enriched functions for target genes and genes negatively correlated with miR-195 include regulation of neuronal and synaptic function, neurogenesis and differentiation, whereas functions of genes positively correlated with miR-195 are enriched in the circulatory system and vasculature development.

**Supplemental Table 3. Gene set enrichment analysis (GESA) studies of EFAD mouse brains with miR-195 over-expression.** We categorized differentially expressed genes (DEGs) between various comparisons: down-regulated genes or up-regulated genes; E4FAD control *versus* miR-195; E3FAD control *versus* miR-195. Most DEGs are enriched in regulation of neuron, synapse, and immune functions. Further GSEA studies suggest the top pathways perturbed by miR-195 over-expression are mitochondrial related pathways.
