# Supplementary figures and images for: MicroRNA-195 rescues ApoE4-induced cognitive deficits and lysosomal defects in Alzheimer’s disease pathogenesis
Source: Mol Psychiatry. 2020 Jul 6;26(9):4687–701. doi: 10.1038/s41380-020-0824-3 (PMC7785685; doi:10.1038/s41380-020-0824-3)

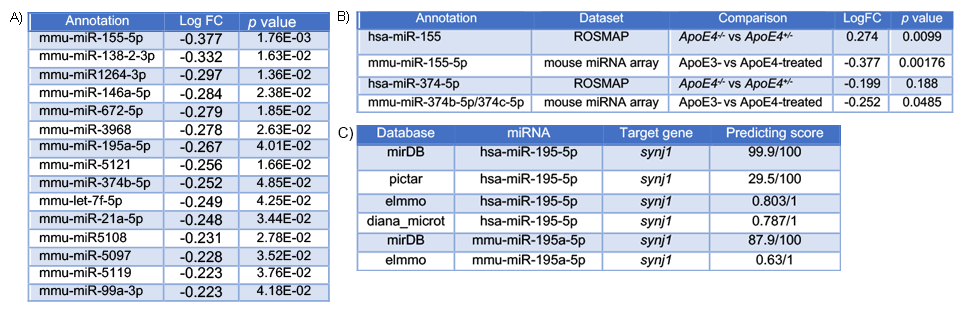

Supplement: Supplementary file 2 — Supplemental Figure 1 [file 41380_2020_824_MOESM2_ESM.tif]

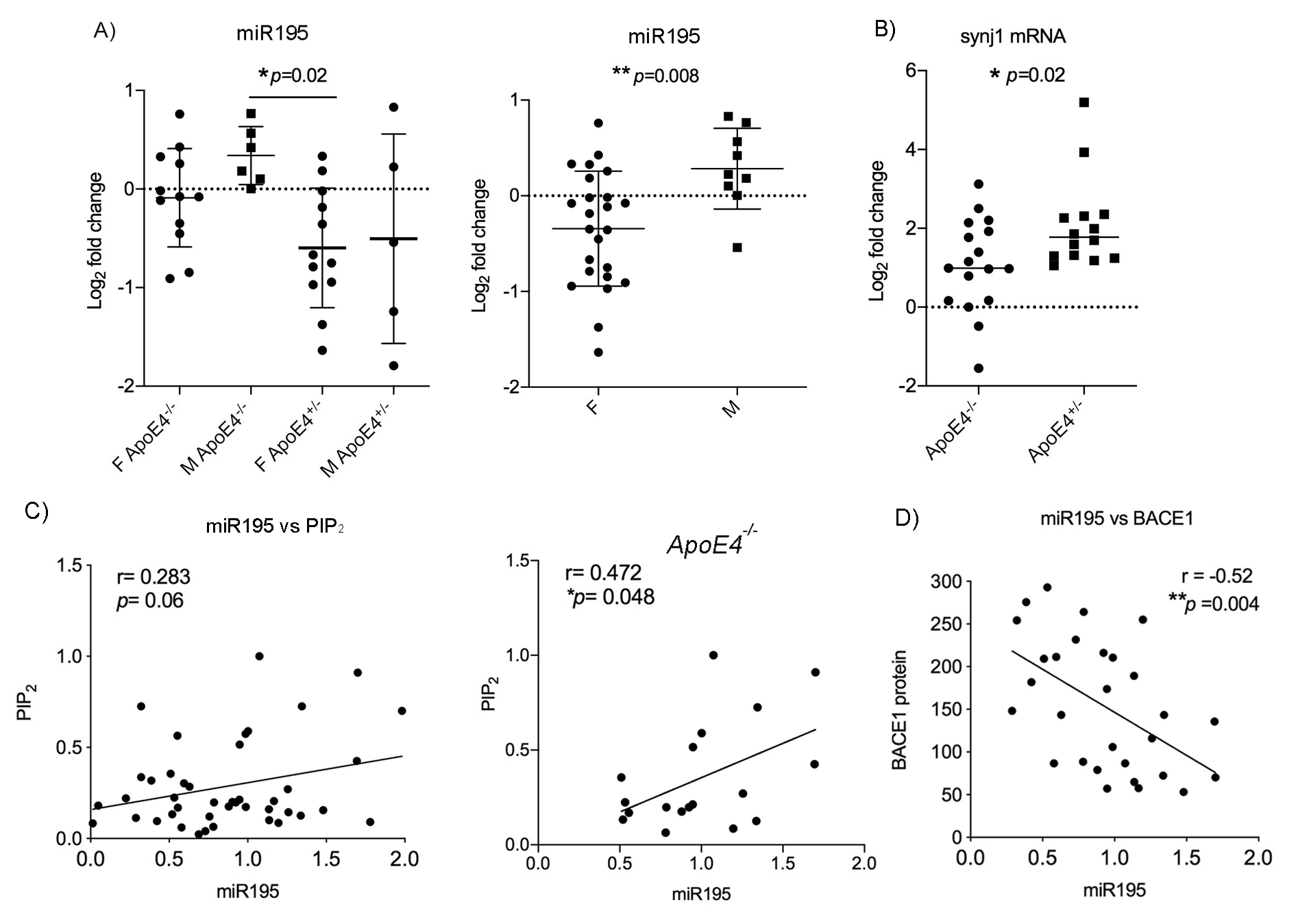

Supplement: Supplementary file 3 — Supplemental Figure 2 [file 41380_2020_824_MOESM3_ESM.tif]

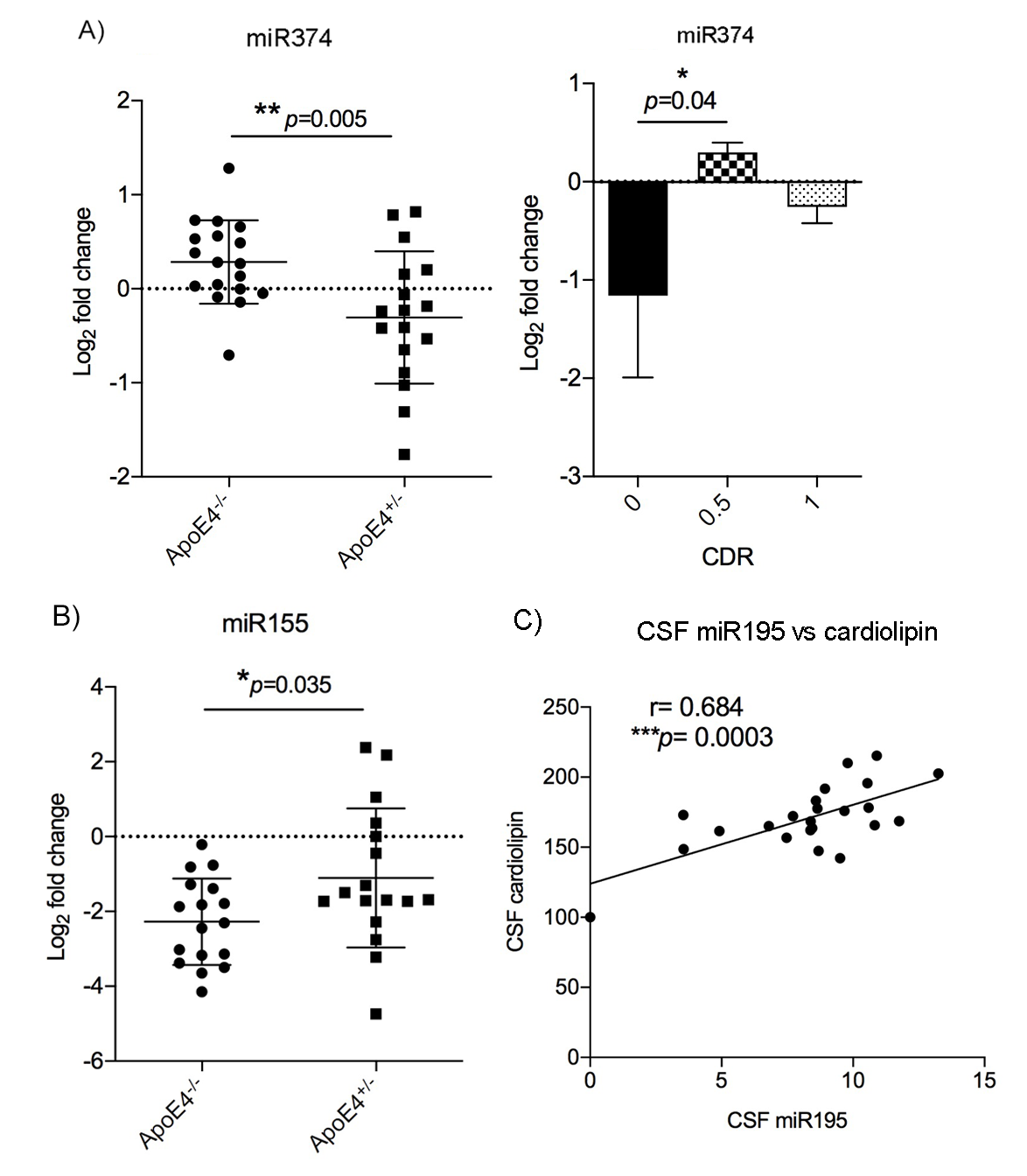

Supplement: Supplementary file 4 — Supplemental Figure 3 [file 41380_2020_824_MOESM4_ESM.tif]

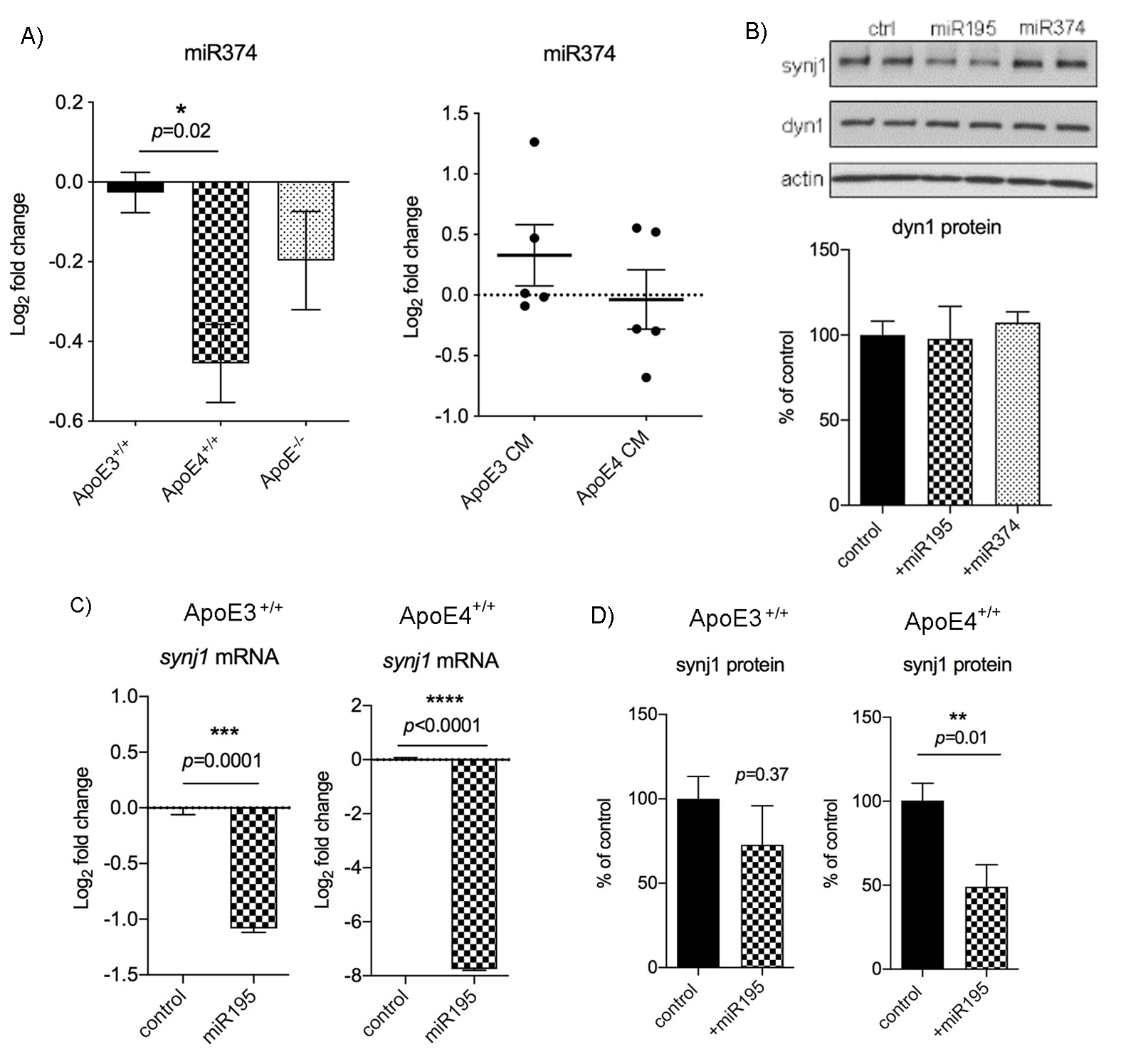

Supplement: Supplementary file 5 — Supplemental Figure 4 [file 41380_2020_824_MOESM5_ESM.tif]

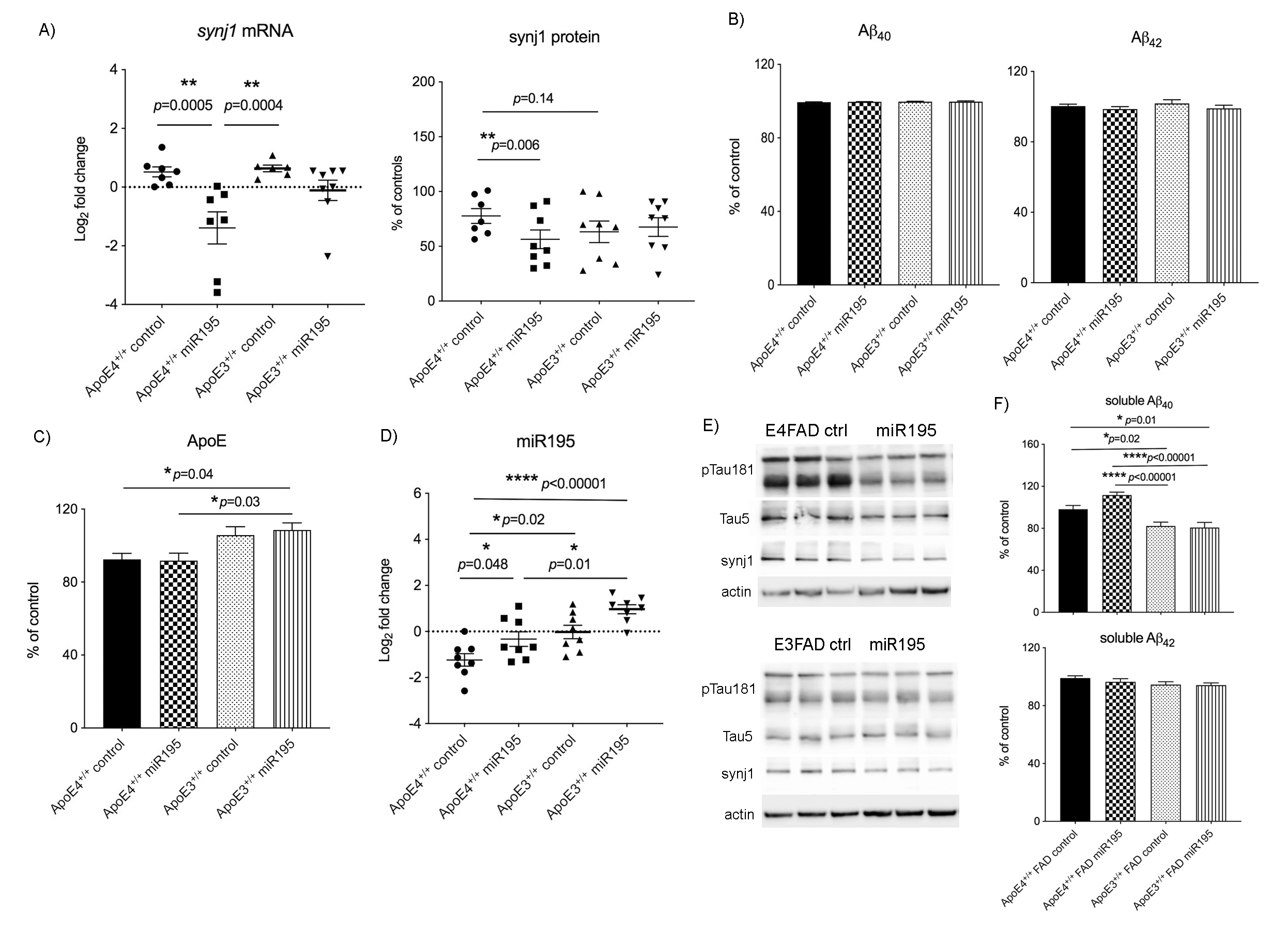

Supplement: Supplementary file 6 — Supplemental Figure 5 [file 41380_2020_824_MOESM6_ESM.tif]

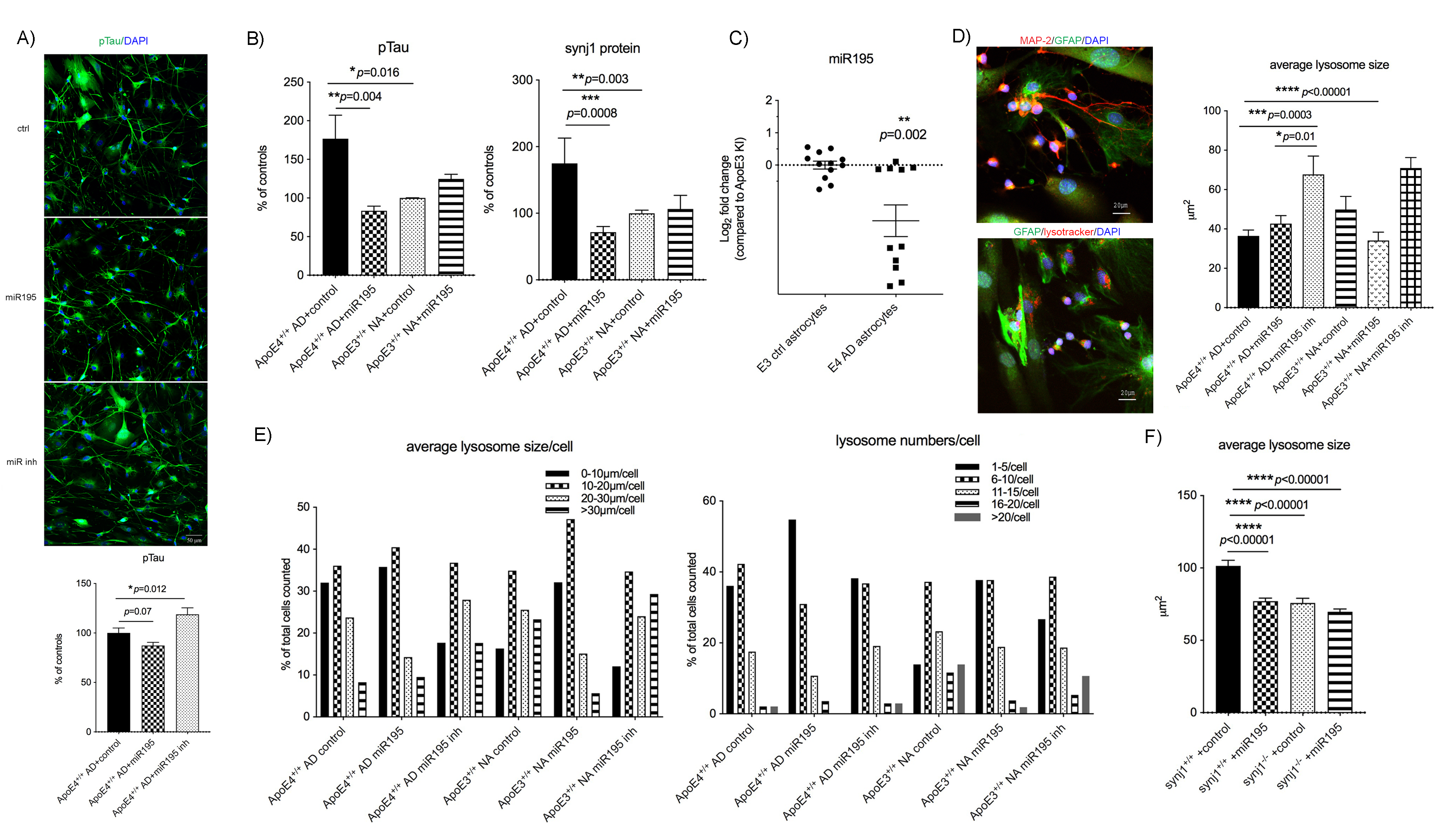

Supplement: Supplementary file 7 — Supplemental Figure 6 [file 41380_2020_824_MOESM7_ESM.tif]

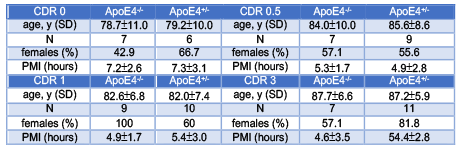

Supplement: Supplementary file 8 — Supplemental Table 1 [file 41380_2020_824_MOESM8_ESM.tif]

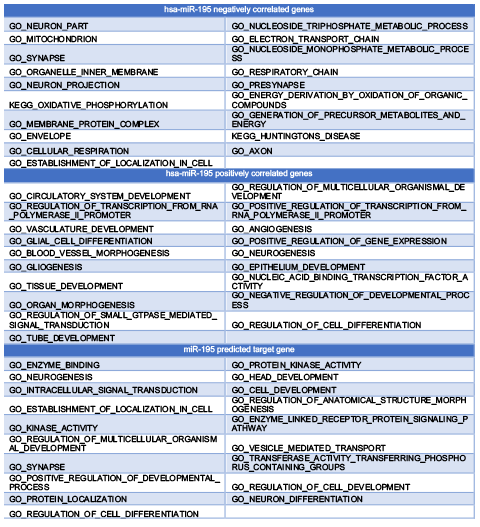

Supplement: Supplementary file 9 — Supplemental Table 2 [file 41380_2020_824_MOESM9_ESM.tif]

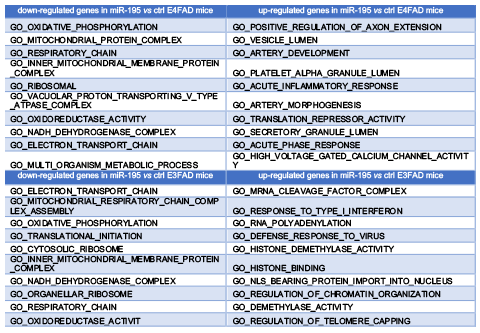

Supplement: Supplementary file 10 — Supplemental Table 3 [file 41380_2020_824_MOESM10_ESM.tif]
